# Bidirectional Interaction Between Chronic Kidney Disease and *Porphyromonas gingivalis* Infection Drives Inflammation and Immune Dysfunction

**DOI:** 10.1155/jimr/8355738

**Published:** 2025-04-17

**Authors:** Karina Adamowicz, Andrea Sofia Lima Ribeiro, Anna Golda, Marta Wadowska, Jan Potempa, Christoph Schmaderer, Hans-Joachim Anders, Joanna Koziel, Maciej Lech

**Affiliations:** ^1^Department of Microbiology, Faculty of Biochemistry, Biophysics and Biotechnology, Jagiellonian University, Cracow, Poland; ^2^Department of Medicine IV, LMU University Hospital, LMU Munich, Munich, Germany; ^3^TUM University Hospital, Technical University Munich (TUM), Munich, Germany; ^4^Department of Oral Immunity and Infectious Diseases, University of Louisville School of Dentistry, Louisville, Kentucky, USA

**Keywords:** CKD, infection, *P. gingivalis*, uremia

## Abstract

**Introduction:** Chronic kidney disease (CKD) is characterized by a decline in renal function, increased mortality, and significant impairments in the immune system and function of immune cells. These alterations are often derived by uremic toxins, which, in turn, modify the immune system's response to infections. Our research investigates the progression of *Porphyromonas gingivalis* (*P. gingivalis*) infection during CKD and its subsequent impact on kidney failure.

**Methods:** We utilized two infectious models, a chamber model representing short-term local inflammation and alveolar bone loss that mimic chronic infection of periodontium, both in conjunction with a CKD model. Additionally, our in vitro studies employed primary macrophages, osteoclasts, and lymphocytes to characterize the immune responses to *P. gingivalis* and pathogen-associated molecular patterns (PAMPs) in the presence of uremic toxins.

**Results and Conclusion:** Our findings demonstrate that uremic toxins, such as indoxyl sulfate (IS), alter responses of macrophages and lymphocytes to *P. gingivalis*. In vivo, CKD significantly enhanced *P. gingivalis* survival and infection-induced alveolar bone loss. The increased distribution of pathogen within peripheral tissues was associated with altered inflammatory responses, indicating that CKD promotes infection. Moreover, *P. gingivalis*-infected mice exhibited a marked increase in renal inflammation, suggesting that the relationship between uremia and infection is bidirectional, with infection exacerbating kidney dysfunction. Furthermore, we observed that infected CKD mice exhibit decreased serum immunoglobulin G (IgG) levels compared to infected mice without CKD, implying that uremia is associated with immune dysfunction characterized by immunodepression and impaired B lymphocyte function.

## 1. Introduction

Chronic kidney disease (CKD) is a medical condition that affects millions of individuals worldwide [1–3]. The patients show progressive loss of kidney function over time, associated with significant physiological and immunometabolic changes [4–6]. One of the major challenges for CKD patients is their increased susceptibility to infections. For instance, studies have shown that over a 7-year follow-up period, 11.7% of all hemodialysis patients and 9.4% of peritoneal dialysis patients experienced at least one episode of septicemia [7]. Additionally, mortality due to sepsis in chronic dialysis patients is 100–300 times higher than in the general population [8]. Notably, infections associated with CKD, such as those related to neuropathy, peripheral vascular disease, and pneumonia, as well as urinary tract, skin, and other soft tissue infections, account for over 40% of all cases [9]. Bacteria such as *Staphylococcus aureus*, *Pseudomonas aeruginosa*, and *Escherichia coli* are commonly isolated as uncontrolled pathogens from the lungs, abdomen, skin/soft tissues, or urinary tracts of uremic patients [10, 11]. Research has shown that mortality due to infections is significantly higher in hemodialysis and peritoneal dialysis patients compared to the general population, with a 14-fold to 16-fold increase specifically for pulmonary infections [9]. This increased susceptibility is linked to several factors, including impaired immune responses, changes in immunological function, and chronic inflammation associated with CKD. As a result, CKD patients are at great risk for acquiring infections, and these infections are often more severe, prolonged, or recurrent, further compromising their health. There is notable dysregulation of various immune cell types, including monocytes, macrophages, and T cells. For instance, blood monocytes in CKD patients show impaired reactive oxygen species (ROS) release, and the phagocytic activity of neutrophils and monocytes is reduced compared to cells from nonuremic controls [12, 13]. Furthermore, end-stage renal disease (ESRD) which represents a severe form of CKD results in an acquired immunodeficiency. This has been referred to the induction of anti-inflammatory cytokines such as IL10 [14]. This increased susceptibility to infections, along with immune cell alterations and an elevated risk of rapid progression of autoimmune disorders, underscores the complex interplay between CKD and immune function [12, 15, 16]. Our recent findings demonstrate that uremic toxins such as indoxyl sulfate (IS) induce ROS release and contribute to low-grade inflammation in macrophages [17]. Additionally, under proinflammatory conditions induced by lipopolysaccharide (LPS), IS significantly elevated the release of tumor necrosis factor α (TNFα), CCL2, and IL10, although it did not substantially affect macrophage polarization. Furthermore, experiments conducted on tubular epithelial cells suggest that IS may induce cellular senescence, potentially contributing to the progression of inflammaging [17].

On the other hand, chronic infections are a critical factor in the progression of organ damage, acting as a persistent source of inflammation that exacerbate tissue injury and degeneration [18–20]. This is attributed to systemic inflammation driven by bacterial endotoxins and immune mediators, which promote endothelial dysfunction, oxidative stress, and fibrosis [21–23]. For instance, in chronic pulmonary disease, infections like *P. aeruginosa* in patients with cystic fibrosis or chronic obstructive pulmonary disease (COPD) further damage lung tissue by perpetuating cycles of inflammation and tissue destruction [24]. Recurrent or chronic urinary tract infections (UTIs) can lead to pyelonephritis and scarring of renal tissues, worsening CKD [25]. Another example is seen in cardiovascular diseases, where *Chlamydia pneumoniae* infections have been implicated in the progression of atherosclerosis, leading to more severe heart damage in patients with pre-existing cardiovascular conditions [26]. While the direct impact of chronic infections on health is well recognized, their role in accelerating the decline of already compromised organs is gaining increasing attention. For example, in diabetic patients, chronic foot infections can lead to systemic inflammatory responses that worsen renal and cardiovascular complications [27]. Similarly, tuberculosis (TB), though primarily a pulmonary infection, can induce renal amyloidosis in patients with CKD, further aggravating kidney damage [28].

*Porphyromonas gingivalis*, a keystone pathogen in periodontal disease, has garnered significant attention for its profound impact on systemic chronic inflammation and its association with various systemic conditions [29]. Unlike many bacteria that remain localized to the oral cavity, *P. gingivalis* demonstrates remarkable adaptability and virulence, enabling it to survive within host tissues and organs beyond its primary niche [30–35]. Evidence suggests that *P. gingivalis* can translocate into systemic circulation, colonize distant organs, and exacerbate pathological processes [36–39]. What sets *P. gingivalis* apart from other pathogens is its arsenal of virulence factors that enable evasion of host immune responses and persistence within host tissues. Among these are its gingipains, a family of proteolytic enzymes that degrade host proteins and modulate immune signaling to create a favorable environment for bacterial survival [40]. A soluble or embed in outer membrane vesicles (OMVs), proteases expand their effectiveness and range of activity by diffusion to surrounded tissues and beyond. Additionally, *P. gingivalis* is adept at subverting host immune pathways. It can manipulate toll-like receptor (TLR) signaling and promote immune tolerance, which prevents its clearance and contributes to chronic inflammation [41, 42]. Once systemic, *P. gingivalis* can invade host cells, such as endothelial cells, macrophages, and dendritic cells, further propagating inflammation and facilitating its dissemination to distant sites [43]. By disrupting the host's immune balance, it contributes to the pathogenesis of cardiovascular diseases, rheumatoid arthritis, diabetes, and neurodegenerative disorders such as Alzheimer's disease since gingipains may directly contribute to neuronal damage [44].

In the present study, we investigated the impact of CKD on in vivo infection with periodontal pathogen *P. gingivalis* using two infectious models: chamber model and oral gavage model of alveolar bone loss [42, 45]. Our results indicate that CKD significantly increased survival and organ distribution of *P. gingivalis*. We also observed a reduction in serum immunoglobulin G (IgG) levels in CKD mice infected with *P. gingivalis* compared to mice without CKD. As a result, the infected CKD mice exhibited markedly accelerated disease progression. Analysis of proinflammatory cytokine and chemokine expression at both the gene and protein levels revealed several alterations in inflammatory processes and homeostasis. In summary, our findings provide experimental evidence that CKD compromises the body's defense mechanisms by sustaining low-grade inflammation and diminishing B lymphocyte responses.

## 2. Materials and Methods

### 2.1. Bacterial Culture

*P. gingivalis* strain ATCC 33277 or W83 (ATCC BAA-308) was cultured anaerobically (90% N_2_, 5% H_2_, and 5% CO_2_) on blood agar or in TSB broth with yeast extract (contains 5 μg/mL hemin, 0.5 μg/mL menadione, and 50 μg/mL L-cysteine) at 37°C. The live bacteria concentrations were adjusted to an optical density (OD) of 1.0 at 600 nm, which corresponds to 10^9^ colony-forming unit (CFU)/mL.

### 2.2. Mice

Female C57BL/6 mice of 6–8 weeks of age were kept in specific pathogen-free conditions and randomly assigned to study groups (*n* = six per each group/experiment). All procedures were approved by the local government authorities (II LKE) in Krakow (reference number: 70/2018, 257/2018).

### 2.3. CKD Model

In this experimental setup, animals were treated to induce the CKD model by intraperitoneally injecting a solution of aristolochic acid I (AAI) salt in PBS at a dose of 5 mg/kg body weight on days 0, 2, 4, 6, 8, and 10. Control animals received only PBS injections [46]. On day 30, a subset of animals from both the AAI and PBS groups was subjected to infection with *P. gingivalis*. The specifics of the bacterial strains, inoculation protocols, and bacterial loads used for infection were detailed in subsequent experimental methods.

### 2.4. Subcutaneous Chamber Model

As previously described [45], a surgical-grade titanium wire coil (diameter 5 mm) was implanted subcutaneously in the dorsolumbar region of each mouse. After a 10-day healing period, 0.1 mL suspension of bacteria cells (2 × 10^8^ CFU/mL) was injected into the chambers, with PBS used as a control. Chamber fluid was collected at various time points for analysis. Bacterial CFU enumeration, myeloperoxidase activity, and cytokine responses were assessed via ELISA. The *P. gingivalis* W83 strain (2 × 10^8^ CFU/mL) was used for the experiments shown in Supporting Information 1: Figure S1. Due to increased mortality and the poor condition of the animals, chamber fluid (10–15 µL) was collected on days 1 and 3, and the experiment was terminated. The *P. gingivalis* ATCC 33277 strain (2 × 10^8^ CFU/mL) was used to generate data for Supporting Information 2: Figure S2. No increased mortality or signs of fatigue were observed with this strain. Chamber fluid (10–15 µL) was collected on days 1, 3, 5, 7, and 10. The experimental groups included the PBS control group, the *P. gingivalis*-infected group (P.g.), the CKD group (AAI), and the CKD-*P. gingivalis*-infected group (AAI + P.g.). Ten days after the initial infection (40 days following the first AAI treatment), the mice were sacrificed, and tissues were collected for organ analysis.

### 2.5. Oral Infection With *P. gingivalis*

Female mice were administered antibiotics (sulfamethoxazole at 0.87 mg/mL and trimethoprim at 0.17 mg/mL) in their drinking water for 10 days followed 2 days without antibiotics. Periodontitis (PD) was induced by administering 0.1 mL bacterial suspension (10^9^ CFU *P. gingivalis* ATCC 33277 strain or PBS for the control group) using a stainless steel feeding needle (AgnTho's). The infection was repeated every other day for a total of five times. Thirty days after the initial infection (60 days following the first AAI treatment), the mice were sacrificed, and tissues were collected for organ analysis and bone loss evaluation. The experimental groups included the PBS control group, the *P. gingivalis*-infected group (P.g.), the CKD group (AAI), and the CKD-*P. gingivalis*-infected group (AAI + P.g.).

### 2.6. Bone Loss Evaluation and Micro-Computed Tomography (Micro-CT) Analysis

Alveolar bone loss was quantified using micro-CT (MILabs, The Netherlands). Biopsies fixed in 4% paraformaldehyde were scanned using a micro-CT system, and 3D images were reconstructed. Linear measurements were taken from the cemento-enamel junction (CEJ) to the alveolar bone crest (ABC) at two sites on the second molars. Data were quantified using PMOD software (PMOD Technologies Ltd., Switzerland).

### 2.7. Histological Analysis

Paraffin sections (2–4 μm) were stained with periodic acid–Schiff (PAS) and immunostaining techniques. Tubular injury, immune cell infiltration (leukocytes including neutrophils, rat antimouse Ly-6 B.2 [Serotec, UK]; macrophages, F4/80 stain [Serotec, Oxford, UK, 1:50], and fibrosis), and fibrosis (Sirius red staining) were scored semiquantitatively or quantified using ImageJ or Photoshop software. Fibrosis was assessed by collagen staining (Sirius red), and fibrosis severity was scored from 1 to 5 based on the percentage of stained area [47]. We analyzed renal tissue sections using both qualitative and semiquantitative methods to evaluate fibrosis, macrophage infiltration, Ki67 expression, and tubular epithelial cell changes. For each section, at least three random high-power fields (HPFs) were evaluated. Fibrosis was assessed using Sirius red staining to highlight collagen fibers and α-SMA staining to identify activated fibroblasts. The percentage of positively stained areas per HPF was quantified using Photoshop software. Specifically, we measured the ratio of the stained areas (collagen and α-SMA fibers) to the total tissue area within each HPF. To further classify the severity of fibrosis, we categorized each kidney into one of five stages based on the stained area ratio: score 1, 0%–5% stained area; score 2, 5%–10% stained area; score 3, 10%–15% stained area; score 4, 20%–25% stained area; and score 5, >25% stained area. This staging provided a standardized semiquantitative fibrosis score. For F4/80 macrophage staining, Ki67 staining, and tubular epithelial cell analysis, we assessed the stained area as a percentage of the HPF using Photoshop. Images were captured in black and white to enhance contrast, and the black-stained regions were isolated by setting a specific threshold in the software. The software calculated the stained area within each field, providing quantitative data (% of stained HPF) for comparison across samples. To ensure accuracy, we reviewed the processed images manually to confirm that staining intensity was comparable across samples and that there was minimal background interference. Fields with excessive background staining or technical artifacts were excluded from analysis to maintain data integrity.

### 2.8. Renal Functional Parameter

Serum creatinine was determined using the Jaffé test and blood urea nitrogen (BUN) with enzymatic test (Diasys, Germany) according to company protocol. Total urinary protein concentration was directly measured using a NanoDrop spectrophotometer with integrated protein measurement software. Urine samples were diluted 1:2–1:10 with distilled water, depending on the protein concentration, to ensure accurate readings within the instrument's detection range. Urinary protein concentration was also quantified using the Bradford assay. A standard curve was generated using bovine serum albumin (BSA), and diluted urine samples were processed to fit within the assay's linear range. Absorbance was measured at 595 nm. For albumin-to-creatinine ratio (ACR), urinary albumin levels were determined using a commercial ELISA kit (Bethyl Laboratories, Inc.), following the manufacturer's protocol. Creatinine concentrations were measured using a creatinine assay kit. ACR was calculated as the ratio of urinary albumin (μg/mL) to creatinine (mg/mL) for a standardized assessment of kidney function.

### 2.9. IgG Level in Serum

IgG-specific antibodies in murine sera were measured by ELISA as described previously [48]. NUNC MaxiSorp ELISA plates (Thermo Fisher Scientific) were coated overnight at 4°C with antimouse IgG-Fc fragment antibody (A90-131A, Bethyl) diluted in carbonate–bicarbonate coating buffer (pH 9.6). Plates were washed three times with PBS containing 0.05% Tween-20 (PBST) and blocked with 1% BSA in PBS for 1 h at room temperature. Serial dilutions of serum samples (1:100 to 1:1,000,000) were added in triplicate and incubated for 2 h at room temperature. After washing, wells were incubated with a horseradish peroxidase (HRP)-conjugated secondary antibody (A90-131P, Bethyl) diluted 1:50,000 in blocking buffer for 1 h. Plates were washed and developed with 100 µL of TMB substrate. The reaction was stopped with 2N H_2_SO_4_, and absorbance was measured at 450 nm using a Sunrise plate reader (TECAN). Data were analyzed to quantify IgG levels in the serum samples.

### 2.10. RNA Extraction, Reverse Transcription, and Quantitative Real-Time PCR

Norgene kit was used to extract total RNA from blood, cells, and tissues stored in RNA later, according to the manufacturer's instructions. cDNA synthesis from 1 μg of RNA was performed using Superscript II Reverse Transcriptase (Thermo Fisher, Germany). qRT-PCR was carried out using a Light Cycler 480 (Roche, Germany). All primers used (Supporting Information 3: Table S1) were purchased from Metabion (Martinsried, Germany).

### 2.11. In Vitro Experiments

Bone marrow–derived macrophages (BMDMs) were differentiated from bone marrow cells, isolated from femurs and tibias of mice, as described [49]. Cells were cultured in RPMI 1640 with 10% FBS and 1% penicillin/streptomycin (P/S), supplemented with 2 ng/mL recombinant mouse M-CSF. For human monocyte–derived macrophages (hMDMs), PBMCs were isolated and cultured in RPMI 1640 with 10% autologous human plasma and 50 μg/mL gentamicin. Cells were differentiated into macrophages over 7 days. For B lymphocyte, A20 cells, a mouse B cell lymphoma line (ATCC, # TIB-208), were cultured in RPMI containing 10% FBS, 1% P/S, supplemented with 0.05 mM 2-mercaptoethanol (Bioshop), and cultivated under standard conditions (37°C and 5% CO_2_). IS in concentration of 60 µg/mL was used for stimulation because this tryptophan metabolite and uremic toxin accumulates during kidney dysfunction. Its precursors, such as indole, are found in bacterial environments, including those of *P. gingivalis*. IS is also present in the urine of mice subjected to the AAI model, reflecting systemic inflammation and uremic conditions (Supporting Information 4: Figure S3).

### 2.12. Flow Cytometry

Cells (RPMI medium including 10% FBS and 1% P/S) were seeded with 1% FBS at 0.5 million cells/well and stimulated with IS (60 μg/mL) (Sigma-Aldrich) for 2 h. Next, cells were stimulated with LPS *P. gingivalis* standard (10 µg/mL) (InvivoGen) and *P. gingivalis* ATCC 33277 (MOI 1:10) for 48 h. The following antibodies were used for flow cytometric analysis: FITC antimouse MHC class II (I-A/I-E) (eBioscience), PE rat antimouse Ig k light chain (BD Pharmingen), and APC rat antimouse CD138 (BD Bioscience). B lymphocytes were analyzed by flow cytometry using a BD LSRFortessa (BD Bioscience) and FlowJo v10.9.0 software. The mean fluorescence intensity was measured in each group.

### 2.13. ELISA Assay

The levels of cytokines, that is, interleukin 6 (IL6), IL2, monocyte chemoattractant protein 1 (MCP1), TNFα in BMDM, hMDM, and in mice serum were assessed using commercial ELISAs (R&D DuoSet ELISA Kits) or CBA Mouse Inflammation Kit (BD Biosciences). For serum cytokine measurements, mice were bled, and serum was isolated by centrifugation. For cytokine quantification in cell culture media, supernatants from cultured BMDM or hMDM were collected. Cytokine levels were assessed according to the manufacturer's protocols.

### 2.14. Myeloperoxidase Assay (MPO)

Neutrophil influx into the chamber (day 1 and day 3) was analyzed by measuring the myeloperoxidase activity by EnzChek Myeloperoxidase Activity Assay Kit (Invitrogen, Carlsbad, CA, USA). Briefly, for peroxidation activity, 50 µL of MPO standards and chamber fluids were mixed with 50 µL of 2× Amplex UltraRed reagent working solution and incubated at room temperature for 30 min, protected from light. The reactions were stopped by adding 10 µL of 10× peroxidation inhibitor to all samples, and any increase in fluorescence was detected using an excitation wavelength of 530 nm and emission at 590 nm.

For cell culture and differentiation, BMDMs were extracted from the femur and tibia. A 0.5 mL Eppendorf tube was modified by piercing its base with an 18G needle, which was then placed into a 1.5 mL Eppendorf tube. The bones were inserted into the 0.5 mL tube, and the setup was centrifuged at 10,000 rpm for 15 s at 4°C. The resulting pellet was gently resuspended in 1 mL of 0.155 M NH4Cl (RBC lysis buffer at room temperature) using slow pipetting, after which an additional 2 mL of the buffer was added. The suspension was left at room temperature for 1 min to allow lysis. To terminate the reaction, the lysis buffer was diluted with 10–20 mL of medium, followed by centrifugation at 1500 rpm for 2 min at 4°C. The cells were washed with fresh medium and centrifuged again under the same conditions. Subsequently, the cell suspension was filtered through a 70 μm cell strainer and centrifuged once more. The final pellet was resuspended in 1 mL of medium, and the cells were counted. The cells were then plated in either 12-well plates (1.5 × 10^6^ cells/well) or 6-well plates (3 × 10^6^ cells/well) using 1–2 mL of Dulbecco's Modified Eagle Medium containing 10% fetal calf serum (or mouse serum), 1% P/S, and rmM-CSF at a concentration of 2 ng/mL. On days 2 or 3, 1–2 mL of fresh medium with rmM-CSF was added to the cultures. On day 5, the medium was replaced with a fresh medium containing rmM-CSF. By day 7, the cells were prepared for stimulation. Osteoclasts were generated from human peripheral blood mononuclear cells (PBMCs) isolated from peripheral blood obtained from the Regional Blood Center, Krakow, Poland, as previously described [50]. The blood center deidentified all the blood materials as appropriate to ensure the confidentiality of the human subjects. Thus, this study adheres to appropriate exclusions from the approval of studies with human subjects. Briefly, PBMCs were isolated from blood containing anticoagulant (sodium isocitrate 3.2%) by Pancoll gradient centrifugation yielding the fraction highly enriched in mononuclear cells. Cells were plated at 3 millions/well in 24-well plates (Primaria, Corning) in 1 mL RPMI1640 (Life Technologies) supplemented with 50 μg/mL gentamicin (Life Technologies), and 10% autological human plasma. After 24 h, nonadherent PBMCs were removed by washing with 1× PBS (Life Technologies), and adherent cells were differentiated to osteoclasts in alpha Modified Eagle's Medium (αMEM, Life Technologies) supplemented with 10% FBS (Life Technologies) and 100 U/mL P/S (Life Technologies) for 14 days in the presence of human RANKL (100 ng/mL, R&D Systems) and human M-CSF (25 ng/mL, R&D System). All media and factors (RANKL, M-CSF, IS [60 µg/mL], and inosine [100 ug/mL]) were replaced twice a week.

### 2.15. Tartrate-Resistant Acid Phosphatase (TRAP) Staining

Osteoclasts differentiated from PBMCs were fixed with 4% formaldehyde for 10 min at room temperature and stained for TRAP using a Leukocyte Acid Phosphatase Staining Kit (Sigma-Adrich) according to the manufacturer's protocol. TRAP-positive cells with more than three nuclei were counted as osteoclasts under microscopic examination.

### 2.16. Statistical Analysis

Data were expressed as mean ± SEM or as mean ± SD as indicated in figure legends. Statistical significance (*p*  < 0.05) was determined using Student's *t*-test for normally distributed data or the Mann–Whitney *U* test for nonparametric data. A *p*-value < 0.05 indicated statistical significance.

## 3. Results

### 3.1. CKD Delays *P. gingivalis* Clearance In Vivo and Increases Bacterial Entrance Into the Blood Circulation and Tissues During PD

To determine the involvement of CKD in the pathogenesis of *P. gingivalis* infection in vivo, we first induced CKD in mice and later, performed the subcutaneous chamber model of infection. To trigger the CKD, mice were treated with six doses of AAI as previously described [46]. Thirty days later, two groups of mice (AAI and PBS treated) were inoculated with *P. gingivalis* W83 2 × 10^8^ CFU/mL (respectively, P.g. group and AAI + P.g. group). We observed increased mortality in the infected groups (Supporting Information 1: Figure S1A). Bacteria counts in *P. gingivalis*-infected and AAI-pretreated and infected groups did not differ at day 1 and day 3 upon infection (Supporting Information 1: Figure S1B), and we did not observe any significant differences in body mass between control- *P. gingivalis*-infected group (P.g.), CKD group (AAI), and CKD-*P. gingivalis* -infected group (AAI + P.g.) as illustrated in Supporting Information 1: Figure S1C. The cytokine levels in serum of mice were determined with flow cytometry (Supporting Information 1: Figure S1D). We did not observe any significant differences between infected mice with and without CKD. We investigated the progression of CKD by assessing inflammation in the renal tissue and observed reduced kidney weight in AAI + P.g. group compared to the AAI group (Supporting Information 1: Figure S1E) and increased infiltration of Mac+ and F4/80+ macrophages in the group of infected mice with CKD (Supporting Information 1: Figure S1F). This indicates that infection accelerates the damage within renal tissue.

To confirm that the observed effect is independent of the *P. gingivalis* strain, we adjusted the number of bacteria and used different strains of *P. gingivalis* ATCC 33277. Thirty days post-AAI injection, two groups of mice were inoculated with *P. gingivalis* ATCC 33277 strain (Supporting Information 2: Figure S2). We did not observe increased mortality or fatigue in the groups, and we performed chamber infection by inoculating mice with 2 × 10^8^ CFU/mL of bacteria. The weight loss in AAI and AAI + P.g. was expected (Supporting Information 2: Figure S2A). Bacteria counts in chambers and tissues of control-PBS group, control-*P. gingivalis*-infected group (P.g.), CKD group (AAI), and CKD-*P. gingivalis*-infected group (AAI + P.g.) revealed no significant differences in the survival of *P. gingivalis* in the CKD-*P. gingivalis*-infected group (until day 7) compared with *P. gingivalis*-infected group (Supporting Information 2: Figure S2B). Interestingly, the number of *P. gingivalis* bacteria in both groups was not significantly different over the first 5 days. As expected, we did not detect any bacteria in the PBS and CKD groups. In addition, MPO activity in chamber fluids did not reveal any significant differences between groups (Supporting Information 2: Figure S2C). We investigated the progression of CKD by assessing chronic inflammation, fibrosis, and regeneration of renal tissue. Mice with AAI-induced nephropathy did not show significantly decreased kidney weight compared to other groups (Supporting Information 2: Figure S2D). This finding was supported by the renal histology (Supporting Information 2: Figure S2E). We did not observe any differences between the control and the infected group. However, we demonstrated increased macrophage infiltration in AAI + P.g. group indicating progression of kidney damage (Supporting Information 2: Figure S2F). We concluded that the infection was mild and did not affect the renal tissue significantly. This contention was supported by proinflammatory cytokine levels in serum of the mice. We did not detect any increased levels of cytokine expression in any group of *P. gingivalis*-infected mice indicating rather mild infection (data not shown). Importantly, we were not able to detect *P. gingivalis* DNA in the renal tissues and urine and in the bloodstream. Together, AAI-treated mice (CKD group) showed only a trend toward delayed clearance of bacteria, while the *P. gingivalis*-infected group demonstrated higher levels of bacterial count in chamber fluids at 24 h postinoculation but without systemic dissemination.

**Figure 1:**
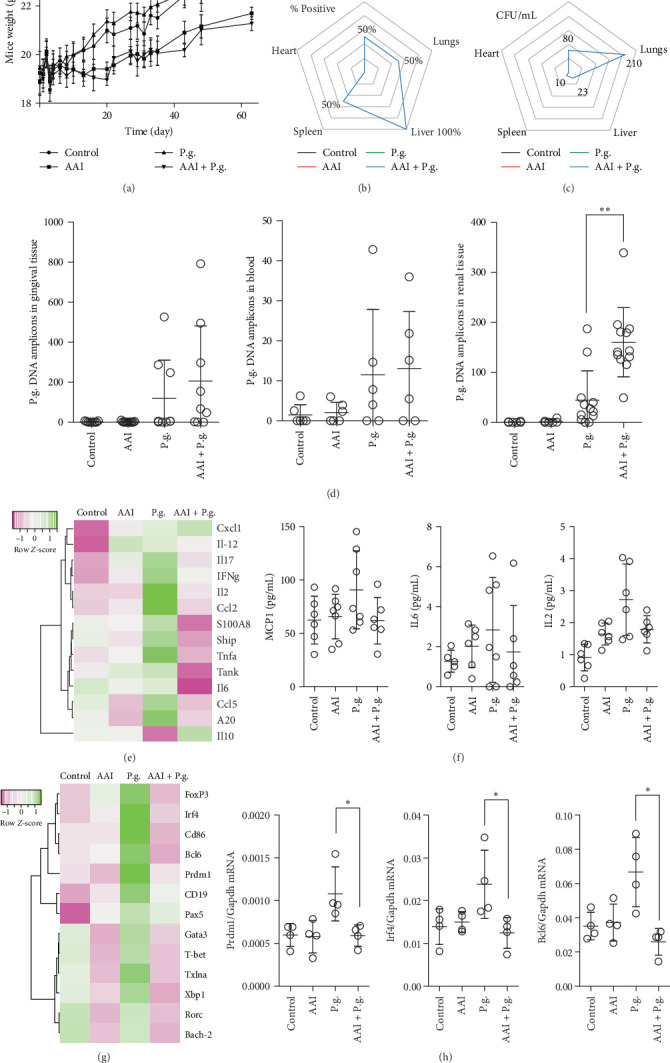
Chronic kidney disease (CKD) affects colonization and survival of *P. gingivalis* strain ATCC 32277 in solid organs upon ligation model. (A) Body mass was monitored for 60 days in all the experimental groups. (B) Survival of *P. gingivalis* was assessed by counting the number of colony-forming units in lung, liver, spleen, heart, and kidney homogenates collected from the control group, CKD group (AAI), *P. gingivalis*-infected group (P.g.), and CKD-*P. gingivalis*-infected group (AAI + P.g.) at day 60 upon CKD induction followed by chronic infection (*n* = 6 per each group). The bacteria grew only in the AAI + P.g. group. The radar chart represents the percentage of mice that tested positive for *P. gingivalis* as assessed by colony counting in the indicated organs. (C) The radar chart shows the number of colonies that grew in mice whose organs tested positive for *P. gingivalis* upon infection: lung (210 colonies), liver (23 colonies), spleen (10 colonies), heart (0 colonies), and kidney (80 colonies). (D) The number of bacteria in gingival tissues and in tissues was assessed by RT-PCR and presented as the number of copies of the detected nucleic acid (based on standard curve). (E) The levels of several innate immunity-related genes were investigated in the spleens of mice. A heat map shows altered genes from expression analysis of preselected transcripts. Genes indicated in green are upregulated, and genes indicated in pink are downregulated to highlight differences between the samples. The rows are *Z*-score scaled. (F) The levels of the indicated cytokines and chemokines was investigated in the blood of mice. (G) The mRNA expression levels of genes during CKD and *P. gingivalis* infection. A heat map shows altered genes from expression analysis of preselected transcripts. Genes indicated in green are upregulated, and genes indicated in pink are downregulated to highlight differences between the samples. The rows are *Z*-score scaled. (H) The levels of several adaptive immunity-related genes were investigated in the spleens of mice. We observed reduction of several gene expressions within the AAI + P.g. group. Data are shown as means ± SD. *⁣*^*∗*^*p*  < 0.05 and *⁣*^*∗∗*^*p*  < 0.01.

Next, we performed a murine model of PD via oral gavage and chose a less virulent *P. gingivalis* ATCC 33277 strain as described in the Materials and Methods section (Figure 1). Thirty days post-AAI injection, two groups of mice were infected with bacteria. We did not observe any increased mortality or differences in weight between the AAI and AAI + P.g. groups (Figure 1A). Bacteria counts in tissues of the PBS group, the *P. gingivalis*-infected group (P.g.), the CKD group (AAI), and the CKD-*P. gingivalis*-infected (AAI + P.g.) group revealed an increased level of *P. gingivalis* in the latter group compared with the P.g. group (Figure 1B,C). As expected, no P.g. was detected in the noninfected PBS and CKD groups. In the AAI + P.g. group, CFUs were quantified in the liver, spleen, kidney, and lungs. Notably, the highest number of colonies was observed in the lungs and kidney. Furthermore, the presence of *P. gingivalis* DNA amplicon was detected in both the P.g. and AAI + P.g. groups in renal tissue, blood, and gingival tissue, underscoring the systemic dissemination of the pathogen (Figure 1D). We observed significantly increased accumulation of *P. gingivalis* DNA in the renal tissues of infected CKD mice. Several cytokines and regulatory factors exhibited downregulation in spleens of CKD-afflicted mice following infection with *P. gingivalis*, as opposed to infected mice without kidney injury. For instance, *IL2*, *IL6*, *IL17*, and interferon-gamma (*Ifn-gamma*) or *S100A8* expression levels were diminished, implying an immune response dysregulation (Figure 1E). Interestingly, the levels of cytokines in the bloodstream remained largely unchanged across groups (Figure 1F), but in the spleens of infected mice, a significant alteration was observed in the levels of inflammatory mediators and mediators relevant to B lymphocytes, especially genes activated during B cell development (Figure 1G,H). In the AAI + P.g. group, these B cell-related genes exhibited a substantial decrease compared to the P.g. group, indicating a profound impact on the immune response and suggesting immune dysregulation in the context of *P. gingivalis* infection combined with CKD.

### 3.2. Gingivalis Infection Affects Serum IgG Levels and Worsens Kidney Function Parameters in CKD Mice

**Figure 2:**
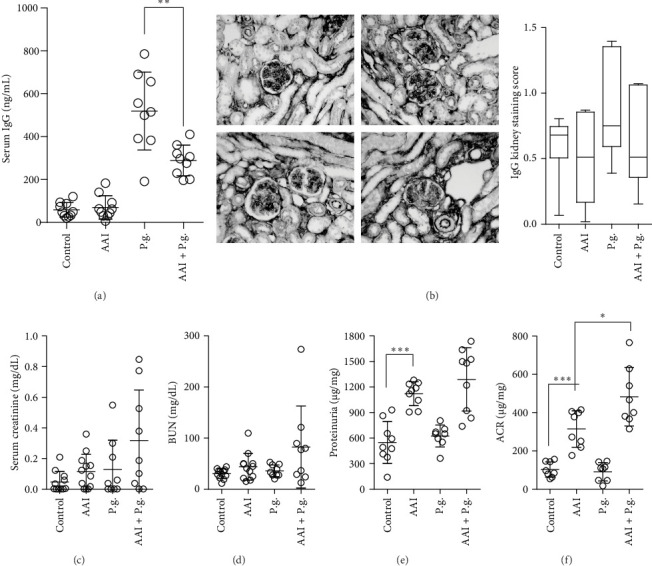
We investigated the serum IgG levels (A) and kidney IgG deposition (B) in the control group, CKD group (AAI), *P. gingivalis*-infected group (P.g.), and CKD-*P. gingivalis*-infected group (AAI + P.g.) at day 60 upon CKD induction followed by chronic infection. Our data indicate that adaptive immune responses (including antibody production) might be affected by chronic kidney disease. (C) Serum creatinine levels, (D) blood urea nitrogen, (E) proteinuria, and (F) ACR were assessed. Data are shown as means ± SD. *⁣*^*∗*^*p*  < 0.05, *⁣*^*∗∗*^*p*  < 0.01, and *⁣*^*∗∗∗*^*p*  < 0.001.

Further, we conducted an analysis of the serum IgG levels and kidney parameters in the four experimental groups. In the AAI + P.g. group, we observed a notable reduction in a serum IgG level compared to the P.g. group, indicating an impact of the combined CKD and *P. gingivalis* infection on the humoral response to *P. gingivalis* antigens (Figure 2A). This result explained the prolonged and accelerated infection in the CKD group since the adaptive response to invading bacteria could be decreased. Interestingly, there were no significant differences in IgG deposition within the kidney tissue among the groups (Figure 2B). To gain a comprehensive understanding of kidney function, we assessed key parameters. Serum creatinine levels did not exhibit a significant increase in the AAI + P.g. group, although a discernible trend was observed. Approximately half of the mice in this group displayed creatinine levels at or above 0.4 mg/dL (Figure 2C). A similar trend was noted in BUN, with roughly half of AAI + P.g. mice demonstrating higher BUN levels (Figure 2D). Moreover, the infected CKD group showed an increase in proteinuria, although this increase did not reach statistical significance (Figure 2E). However, it is noteworthy that the ACR was significantly elevated in the AAI + P.g. group when compared to the AAI group (CKD group) (Figure 2F). Importantly, proteinuria and ACR were significantly increased in the CKD group, confirming kidney damage in aristolochic acid treated group (AAI). In summary, the introduction of infection on top of CKD led to a substantial increase in ACR, manifested by a more severe impairment of kidney function.

### 3.3. Gingivalis Infection Accelerates the Progression of Renal Inflammation and Fibrosis

**Figure 3:**
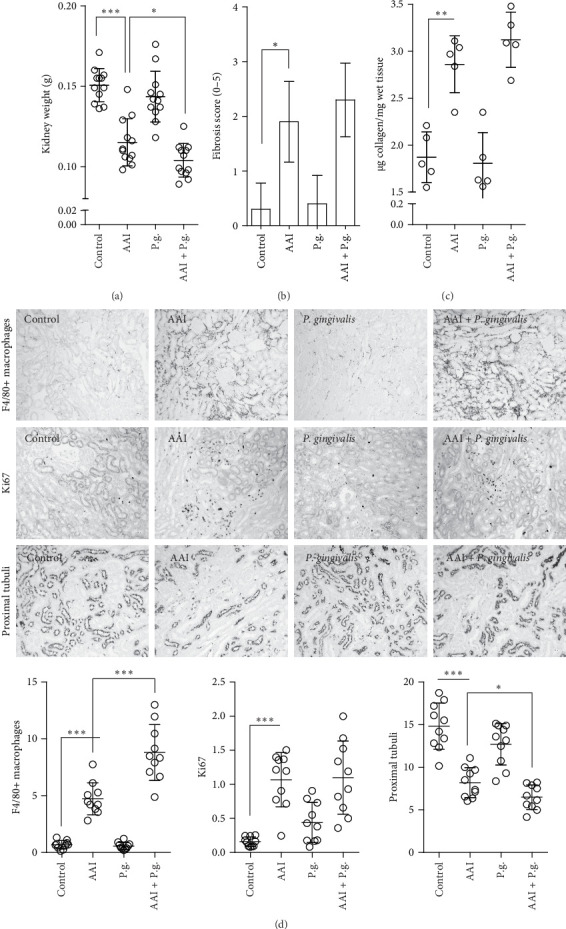
Differences between the control groups, CKD group (AAI), *P. gingivalis*-infected group (P.g.), and CKD-*P. gingivalis*-infected group (AAI + P.g.) in (A) kidney weight and (B) fibrosis scored based on Sirius red staining and (C) collagen within the kidney tissue. (D) The treated and control kidneys from all groups were stained for interstitial macrophages (F4/80), proliferation marker (Ki67), and proximal tubules to illustrate kidney inflammation and regeneration. Quantification was performed in Photoshop as a percentage of positively stained high-power field (data not shown). Differences between groups of mice in F4/80+ macrophages, Ki67, and proximal tubules staining. Scale bar = 50 µm. Data are shown as means ± SD. *⁣*^*∗*^*p*  < 0.05, *⁣*^*∗∗*^*p*  < 0.01, and *⁣*^*∗∗∗*^*p*  < 0.001.

Histopathological analysis revealed pronounced renal fibrosis, macrophage infiltration, and tissue reorganization upon AAI treatment. Tubular compartment loss was evident, with staining indicating a marked reduction in tubular structures in the AAI group compared to controls indicating chronic kidney damage (Figure 3). Mice with AAI-induced CKD showed significantly decreased kidney weight compared to other groups confirming the presence of kidney fibrosis (Figure 3A). We assessed loss of functional tissue using both a fibrosis score and the measurement of micrograms (µg) of collagen per milligram (mg) of wet tissue (Figure 3B,C). Although the fibrosis score showed minimal differences between the CKD and AAI + P.g. groups, the AAI + P.g. group exhibited a slightly higher amount of collagen per mg of wet tissue. This suggests that while full fibrosis may not have developed, there are early signs of collagen accumulation in the AAI + P.g. group. The staining patterns observed in proximal tubules showed the areas of structural loss characterized by reduced tubular density, presence of atrophic tubuli, and increased number of atubular glomeruli in CKD and CKD *P. gingivalis*-infected groups (Figure 3D). The quantification showed the reduction in percentage of tubular area. A significant increase in F4/80+ macrophages was observed in the interstitial regions of the kidney in CKD-induced mice compared to controls as well as AAI + P.g. group compared to P.g. group, suggesting heightened inflammatory activity (Figure 3D). Macrophages were predominantly in tubulointerstitial regions and in fibrotic areas. Increased proliferation of Ki67+ cells, indicating active cellular turnover, was probably associated with inflammatory, reparative, or fibrotic responses since they represented a similar pattern to one found in macrophage staining. These signs of chronic kidney damage in the AAI group were enhanced by the infection, that is, increased infiltration of F4/80+ macrophages (Figure 3D). Moreover, AAI-treated and infected mice exhibited a significant loss of proximal tubule as evidenced by *Lotus tetragonolobus* lectin staining compared to the other groups (Figure 3D). All the above-mentioned findings indicate more severe renal inflammation and damage in the AAI + P.g. group (CKD *P. gingivalis*-infected mice).

Since the infiltration of macrophages into the kidney was significant in the AAI and AAI + P.g. groups (Figure 3), we evaluated the activation of these cells upon contact with pathogen to assess the additional inflammatory effects. Our investigation into stimulation of macrophages with *P. gingivalis* revealed their active participation in the inflammatory response (Supporting Information 5: Figure S4). We employed a primary mouse (Supporting Information 5: Figure S4A) and human (Supporting Information 5: Figure S4B) macrophages, subjecting them to stimulation with *P. gingivalis* and *P. gingivalis* + IS over both short (4 h) and extended (24 h) time points. In the short-term (4 h), discernible differences were noted in the response of human macrophages stimulated with *P. gingivalis* + IS, suggesting a potentially delayed response to the stimuli. However, the longer 24-h time point revealed a substantial and equally significant increase in macrophage activation upon *P. gingivalis* infection (independent of uremic toxin presence), marked by a heightened production of IL6 and TNFα. This observation is of particular significance, as it underscores the potential for a robust inflammatory response in the uremic kidney where both *P. gingivalis* is present and macrophage infiltration is augmented. The amplification of inflammation may consequently lead to more pronounced tissue damage.

### 3.4. Uremic Toxin Alters B Lymphocyte Responses and Development

**Figure 4:**
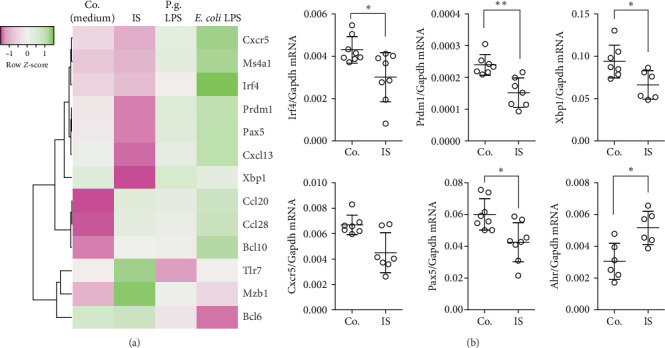
(A) Expression levels of several adaptive immunity-related genes were investigated in the A20 lymphocytes stimulated with indoxyl sulfate (IS), LPS from *P. gingivalis*, or LPS from *E. coli*. We observed reduction of several gene expressions within upon IS-stimulated group. A heat map shows altered genes from expression analysis of preselected transcripts. Genes indicated in green are upregulated, and genes indicated in pink are downregulated to highlight differences between the samples. The rows are *Z*-score scaled. (B) Expression levels of selected adaptive immunity-related genes were investigated in the A20 lymphocytes stimulated with IS. Ahr expression was used as a control. Data are shown as means ± SD. *⁣*^*∗*^*p*  < 0.05 and *⁣*^*∗∗*^*p*  < 0.01.

Intrigued by the reduced antibody levels observed in the AAI + P.g. group, we focused our investigation on B lymphocytes. Specifically, we stimulated these cells with IS, one of the prominent uremic toxins, to assess its impact on genes responsible for B cell function and the development of plasma cells. IS and its precursor indole are present in the blood and tissues of CKD patients [51, 52]. Moreover, gram-negative bacteria including *P. gingivalis* contain tryptophanase, which is responsible for indole production (Supporting Information 4: Figure S3) [53]. Consequently, chronic infection in CKD patients is associated with the constant presence of this uremic toxin [54–56]. We stimulated a B lymphocyte cell line (A20) with IS, LPS from *P. gingivalis* (LPS P.g.) or LPS from *E. coli* (LPS *E. coli*) (used as a positive control) to assess the expression of genes responsible for B cell development and the activation features within the cell line (Figure 4A). As expected, we observed the increased activation of cells upon *P. gingivalis* LPS and even more significant effects with *E. coli* LPS as indicated in the heat map. Upon stimulation with IS, we observed significant decreases in the expression of key genes associated with B cell function and plasma cell development, including *Irf4*, *Prdm1*, *Xbp1*, *and Pax5* (Figure 4B). The results were supported by the flow cytometry analysis of B lymphocyte cell line stimulated with *P. gingivalis* LPS with or without uremic toxin (Supporting Information 6: Figure S5). These findings suggest that IS can negatively affect the regulation of genes essential for B lymphocyte function. To ensure that the cells were metabolically active and functioning adequately, we utilized the aryl hydrocarbon receptor (*Ahr*) as a control (Figure 4B). Ahr is known to respond to IS, and its expression is upregulated upon IS stimulation.

**Figure 5:**
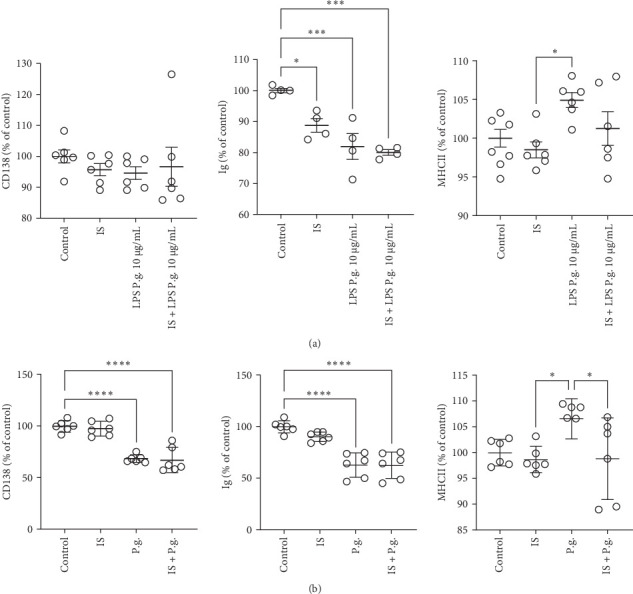
B lymphocyte cell antibody production during uremia condition and infection. B lymphocyte cells were stimulated with IS toxin in the presence/absence of (A) LPS *P. gingivalis* or (B) *P. gingivalis* ATCC 33277 for 48 h. For evaluation of the antibody production, cells were stained for CD138, Ig, and MHCII and were analyzed by flow cytometry. (A) CD138 expression did not change between the groups after stimulation with IS, *P. gingivalis* lipopolysaccharide (LPS *P. gingivalis*), IS + LPS *P. gingivalis*, and the control group. Ig production was reduced in the presence of IS and even more reduced in the presence of IS + LPS *P. gingivalis*. The results demonstrated that there were no significant differences observed among the IS + LPS *P. gingivalis* and LPS *P. gingivalis* groups in MHCII expression. (B) CD138 expression decreased after *P. gingivalis* and IS+ *P. gingivalis* stimulation, the same as Ig antibody production. The expression level of MHCII increased after *P. gingivalis* stimulation compared to the IS and IS + *P. gingivalis*. All data represent mean values ± SD. *⁣*^*∗*^*p*  < 0.05, *⁣*^*∗∗∗*^*p*  < 0.001, and *⁣*^*∗∗∗∗*^*p*  < 0.0001.

Furthermore, we employed costimulation experiments to simulate uremic conditions. Specifically, we subjected B lymphocytes to costimulation with IS and LPS from *P. gingivalis*, as well as IS and *P. gingivalis*, to mimic the complex immune interactions within the context of uremia and chronic infection. In our investigation, we assessed CD138 and major histocompatibility complex II (MHCII) expression, as well as Ig antibody production. Statistical analyses showed no differences in CD138 expression among the experimental groups, including B lymphocytes stimulated with IS, *P. gingivalis* LPS (LPS P.g.), IS + *P. gingivalis* LPS, and the control group (Figure 5A). We also did not observe differences between B lymphocytes stimulated with live *P. gingivalis* and with uremic toxin combined with the live pathogen (IS + P.g.) as illustrated on Figure 5B. However, the presence of IS toxin resulted in a notable reduction in Ig antibody production when compared to the control group. Subsequently, an assessment of MHCII expression was performed following stimulations. The results demonstrated that there were no significant differences observed among the IS + LPS P.g. and LPS P.g. groups (Figure 5A). Nevertheless, a discernible trend was observed, indicating reduced expression and activation levels when B lymphocytes were subjected to the combined stimulation of LPS from *P. gingivalis* and uremic toxin. We observed a significant reduction in MHCII activation in the group stimulated when uremic toxin combined with live pathogens compared to cells stimulated with *P. gingivalis* alone (Figure 5B). Moreover, significant reductions in B cell-relevant proteins, such as CD40, TACI (CD267), or BAFF receptor (CD268), as assessed by flow cytometry, were exclusively observed within the intracellularly stained PAX5-positive population (Supporting Information 6: Figure 5). In summary, results shown in Figures 4 and 5 outline our investigation into the effects of IS on B lymphocytes, shedding light on the altered expression of critical genes, and highlights the fact that uremic conditions affect B lymphocyte responses. However, we were not able to demonstrate the phenotype of the cells using flow cytometry. IS modulates the response to *P. gingivalis* as indicated by the MHCII expression.

### 3.5. CKD Induces Inflammatory Bone Resorption in Bacteria-Induced PD

**Figure 6:**
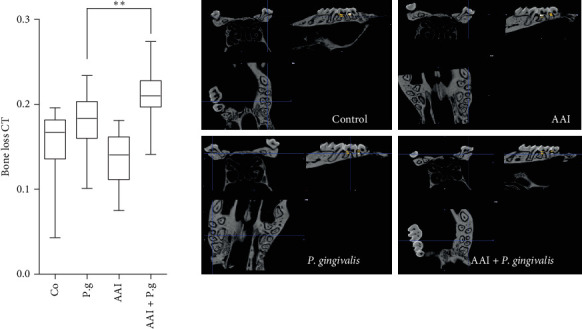
Bone loss following CKD and *P. gingivalis* infection ATCC 32277. Quantification of *P. gingivalis* induced bone loss in wild-type mice with CKD. Exemplary images of sectional micro-computed tomography measurements of the distances from the cementoenamel junction to the alveolar bone crest (CEJ–ABC) on the buccal side at day 60. Results are the mean ± SD. *⁣*^*∗∗*^*p*  < 0.01.

**Figure 7:**
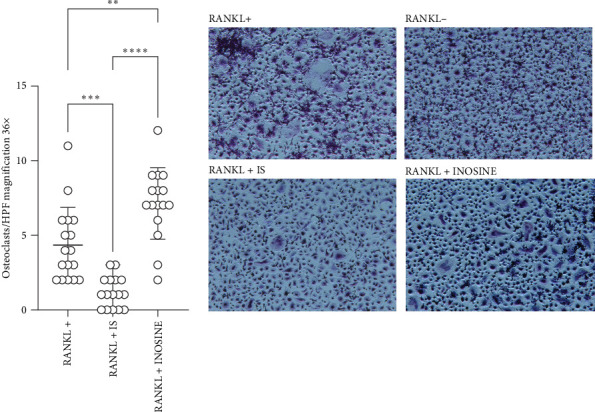
Indoxyl sulfate (IS) but not inosine inhibits RANKL-induced differentiation of PBMCs into osteoclasts. To induce differentiation, PBMCs were cultured for 14 days in the presence of RANKL (50 ng/mL) and M-CSF (25 ng/mL), IS (60 ug/mL), and inosine (100 ug/mL). Osteoclasts were stained for tartrate-resistant acid phosphatase (TRAP). The number of multinucleated osteoclasts was quantified in TRAP-stained samples. Pictures of osteoclasts in the presence of RANKL+, RANKL− (negative control), and uremic toxins: IS and inosine. Data are shown as means ± SD. *⁣*^*∗∗*^*p*  < 0.01, *⁣*^*∗∗∗*^*p*  < 0.001, and *⁣*^*∗∗∗∗*^*p* < 0.0001.

*P. gingivalis* is a late colonizer of oral biofilm that grows in an anaerobic environment of subgingival pockets. Therefore, we further analyzed whether CKD affects periodontal injury and tissue rearrangement in vivo. The mice with preinduced CKD were compared with those inoculated with *P. gingivalis* only, as well as with two control groups (CKD and PBS groups). The AAI + P.g. group demonstrated significantly enhanced alveolar bone loss as evidenced by computerized micro-CT scan (Figure 6). Moreover, we investigated the functional properties of osteoclasts differentiated in the presence of uremic toxins: IS and inosine (Figure 7). PBMC cells were differentiated to osteoclasts with RANKL, incubated with IS or inosine and next characterized for development of specialized bone cells that typically have multiple nuclei [50]. Strikingly, the introduction of IS significantly hindered the formation of TRAP-positive multinucleated osteoclasts. Since osteoclasts play a pivotal role in maintaining the dynamic balance of bone remodeling and the regulation of calcium homeostasis, it remains unsolved whether IS will affect the skeletal adaptation and repair as an additional factor except for infection. On the other hand, the addition of inosine appears to facilitate the differentiation of cells into osteoclasts. This suggests that various uremic toxins and metabolites during uremia, possibly accompanied by infection, may have different effects on osteoclastogenesis.

## 4. Discussion

Despite increasing knowledge and therapeutic advances, the number of patients with CKD continues to grow worldwide. The postinfectious kidney damage includes direct invasion of pathogens or deposition of the antigen–antibody complex by immunological reaction. As to renal dysfunction induced by systemic bacterial infection, some cases of poststreptococcal glomerulonephritis, methicillin-resistant *S. aureus* (MRSA)-related glomerulonephritis, typhus, cholera, *E. coli* infections, and infectious endocarditis are known to cause acute renal failure [57–59] which might abet the development of CKD [60, 61]. Not much is known about effects of local infections and dysbiosis on the progression of kidney disease. Likely, any local (also asymptomatic) infection or inappropriate pathogenic tissue flora is a significant source of potential immune-stimulatory bacterial products which may be responsible for progression of the renal disease. Patients with local pathogen overgrowth and loss of symbiotic flora may be prone to the progression of CKD and development of ESRD. The immune responses of the CKD patients tissues might be significantly impaired and insufficient to fight infections. Therefore, the prevention of progressing microbial infection associated with exaggerated inflammation might be crucial to prevent shift from the early to the end stage CKD.

Our experimental studies focused on periodontal infection and CKD. We demonstrated that the low-grade systemic chronic inflammation originating from local infection such as PD is a risk factor for CKD progression. Our study showed that bacteria and their products enter the blood circulation and tissues of different organs, including kidney. A healthy immune system has the capacity to eliminate pathogens efficiently; however, individuals suffering from CKD respond to such invasion with elevated renal damage. Our data supports the findings of other groups. PD has been already considered as a risk factor for a number of systemic diseases including CKD [62–66]. Latest meta-analysis found that CKD was associated to higher risk of PD (MH-OR = 2.36 [95% C.I. 1.25, 4.44]; *p*=0.008), higher mean CAL (WMD = 0.41 mm [95% C.I. 0.22, 0.60]; *p*  < 0.0001), and mean PPD (WMD = 0.25 mm [95% C.I. 0.03, 0.47]; *p*=0.02) compared to healthy individuals. The same analysis demonstrated that severe CKD (stages 4–5 vs. 2–3) resulted at higher risk of PD (MH-OR = 2.21 [95% C.I. 1.07, 4.54]; *p*=0.03) [67]. Importantly, our data delivered the experimental evidence supporting this concept. Moreover, an increasing body of evidence has established PD as a potential risk factor for numerous systemic conditions, such as atherosclerosis, cancer, and Alzheimer's disease [68, 69]. Our study provides experimental evidence corroborating previous findings that *P. gingivalis* significantly participate in the development of chronic diseases.

The concept of inflammation-induced immunodepression resulting in impaired B cell function, reduced antibody production, altered B cell populations, and compromised development of memory B cells is known [69–72]. These changes can have far-reaching consequences for the overall immune response and the ability to combat infections. Already in the 60 s, immunodeficiency was a well-established observation in individuals with uremia [73]. It has been proposed that abnormalities at both the cellular and humoral levels contribute to this compromised immune reactivity. Our study demonstrates that the survival of *P. gingivalis* and the spreading of infection were significantly enhanced upon uremia. Moreover, the IgG production in infected uremic mice was reduced compared to mice infected without previous kidney injury. This fact is of particular significance in light of investigations that highlighted a notably high percentage of nonresponders among uremic patients following vaccination with hepatitis B antigen [74], as well as other vaccines [75, 76]. Uremia impairs antigen-specific adaptive immunity as evident from insufficient priming of antigen-specific T/B cell responses upon vaccination and results in decreased response to vaccination and a depletion of naïve and memory T cell and B cells [77]. The above-mentioned findings were linked to dysfunction within the peripheral T and/or B lymphocyte populations and antibody production. We could not determine whether the immunodeficiency comes from an overall reduction in the production of IL2 by all lymphocytes or, alternatively, from a diminished fraction of IL2 producers within the particular T cell population. Nevertheless, we observed lower IL2 levels in infected, kidney-injured animals.

An expanding body of evidence suggests that IS may play a substantial role in the progression of CKD. In vitro studies have revealed that IS exerts influences on tubular cell biology, resulting in heightened levels of oxidative stress, inflammation, and fibrosis [78–81]. Animal models further support these findings by demonstrating that IS can expedite CKD progression through nephrotoxic effects [82, 83]. Moreover, serum levels of IS have been associated with various chronic conditions [84, 85]. Our experimental results shed light on the significant role of IS on B lymphocyte development and function. Our data suggest that IS has the potential to impair B cell function and the production of antibodies, affecting the overall humoral immune response. Consequently, in individuals with CKD, the compromised B cell function contributes to an increased susceptibility to infections and other immune-related complications [86]. Our data support this concept and demonstrate the gene expression changes in B cells stimulated with IS. These genes are key players in orchestrating B cell development and plasma cell differentiation. For instance, PAX5 is a transcription factor essential for early B cell development. It maintains B cell identity by repressing genes associated with other cell lineages. It also regulates the expression of genes involved in Ig gene rearrangement. Additionally, significantly reduced transcript encodes interferon regulatory factor 4 (IRF4) which plays a vital role in the differentiation of B cells into plasma cells and regulates the expression of genes involved in antibody production and class switching. Other transcripts reduced upon IS stimulation include PRDM1 (Blimp-1) and X-box binding protein 1 (XBP1)—genes promoting development of antibody-producing plasma cells and protein secretion.

B cell lymphopenia and alteration in B cell transcriptomic have been previously documented in patients with ESRD [87–93]. Furthermore, studies in children with chronic renal failure have demonstrated a reduced population of CD5+ innate B cells and CD27+ memory B cells [88]. Another study conducted by Pahl et al. [94] revealed a substantial reduction in all B cell subpopulations in ESRD patients, except for transitional cells. The study by Pahl et al. [94] showed that plasma levels of IL7, a cytokine facilitating the conversion of pre-B cells to B cells, were elevated in ESRD patients. They concluded that it is more likely that transitional B cells may become resistant to differentiation and survival signals in the uremic milieu since their study did not identify differences in the proportions of apoptotic B cells between ESRD patients and control subjects [94]. In contrast, Fernández-Fresnedo et al. [92] reported increased apoptosis of B cells in patients with chronic renal failure.

While the precise mechanisms behind the observed immunosuppression during uremia remain elusive, our findings align with the existing literature, indicating a noticeable reduction in antibody production among CKD mice. It is important to mention that ESRD is the final stage of CKD, representing a nearly complete loss of kidney function. In contrast to other studies in the field, our research did not focus on the characterization of B cell populations. Instead, we honed in on the pivotal relationship between infection and its reciprocal influence on uremia. Our investigation unveiled a compelling connection between *P. gingivalis* infection and CKD, demonstrating that the presence of this pathogen exacerbated kidney inflammation, resulting in a heightened severity of proteinuria. Equally remarkable was our discovery that uremia significantly facilitated the propagation of the *P. gingivalis* infection. This novel perspective sheds light on a previously overlooked dynamic within the realm of infectious diseases and renal health, offering fresh insights into the intricate interplay between bacterial infections and kidney-related conditions. This investigation explored immunometabolic changes associated with CKD and their profound effects on the risk, course, and outcomes of infections in CKD individuals. By presenting the experimental evidence for these homeostatic shifts, we hope to shed light on potential therapeutic targets and strategies for CKD-related complications, including infections. Exploring this problem is essential for improving patient care and developing innovative interventions to mitigate the burden of CKD on affected individuals.

## Data Availability

All data generated and analyzed during this study are included in this article and its Supporting Information files. Further inquiries can be directed to the corresponding author.

## Supporting Information

Supporting Information 1 Figure S1: CKD did not affect inflammatory response to *P. gingivalis* in vivo. Strain W83 was injected into the subcutaneous chambers in mice. (A) First, we evaluated the survival and health status of the mice. Morbidity and mortality rates were monitored daily and compared for all the groups after bacterial infection. (B) The levels of live *P. gingivalis* in chamber fluids did not differ significantly. (C) The body mass was monitored and quantified as % of body weight loss at day 7 postinfection. (D) Quantification of serum cytokines in the control group, CKD group (AAI), *P. gingivalis*-infected group (P.g.), and CKD-*P. gingivalis*-infected group (AAI + P.g.). (E) Kidney weight was quantified in all four groups of mice. (F) The treated and control kidneys from all groups were stained for interstitial (F4/80+) and glomerular (Mac2+) macrophages. Quantification was performed in Photoshop as percentage of positively stained high-power field (HPF) or quantified by counting as indicated in the figure. Data are shown as means ± SD. *⁣*^*∗*^*p* < 0.05, *⁣*^*∗∗*^*p* < 0.01, and *⁣*^*∗∗∗*^*p* < 0.001.

Supporting Information 2 Figure S2: CKD did not significantly affect response to *P. gingivalis* in vivo. Strain ATCC 33277 was injected into the subcutaneous chambers in mice. We observed 100% survival and no significant changes in overall health status. (A) We evaluated the body mass for 60 days. (B) The levels of live *P. gingivalis* in chamber fluids showed prolonged survival of the pathogen within CKD-*P. gingivalis*-infected group (AAI + P.g.). (C) We did not observe significant changes in MPO activity in chamber fluids between P.g. and AAI + P.g. groups at days 1 and 3. (D) Kidney weights and (E) fibrosis scores showed no differences between CKD group (AAI) and CKD-*P. gingivalis*-infected group (AAI + P.g.). (F) Kidneys from all groups were stained for T cells (CD3+), interstitial macrophages (F4/80+), proliferation marker (Ki67), and proximal tubuli to illustrate kidney inflammation and regeneration. Quantification was performed in Photoshop as percentage of positively stained high-power field (HPF). Data are shown as means ± SD. *⁣*^*∗*^*p* < 0.05 and *⁣*^*∗∗∗*^*p* < 0.001.

Supporting Information 3 Table S1: Primer list.

Supporting Information 4 Figure S3: (A) The indican/indoxyl sulfate levels in urine were measured with MAK128 (Sigma) kit at 480 nm. (B) Since indole is a precursor of indoxyl sulfate and is produced as a metabolic intermediate during the breakdown of the amino acid tryptophan by bacteria, we measured the production of indole in *P. gingivalis* strains used in the experiment; we used uropathogenic *E. coli* as a positive control. (C) Bacterial colonies grown on agar plates containing Luria–Bertani (LB) and blood agar plate (BAP) medium after inoculating peritoneal fluid. Colonies were counted to assess the microbial load in the peritoneal fluid sample 3 days after induction of kidney injury with AAI.

Supporting Information 5 Figure S4: Macrophage cytokine production upon indoxyl sulfate and infection with *P. gingivalis*. (A) and (B) Bone marrow–derived macrophages (BMDM) were incubated with 60 μg/mL IS for 4 h, infected for 2 h, and washed; cytokine production was measured after 4 and 24 h. We observed a significant increase in the production of cytokines following an infection with P.g. Indoxyl sulfate delays the BMDMs' response to *P. gingivalis* infection; however, the levels of cytokines in P.g. and P.g. + IS groups at 24 h postinfection are not significantly different. (C) and (D) Human monocyte–derived macrophages (hMDMs) were incubated with 60 μg/mL IS for 4 h, infected for 2 h, and washed; cytokine production was measured after 4 and 24 h. We observed a significant increase in the production of cytokines following an infection with P.g. Indoxyl sulfate does not affect hMDMs' response to *P. gingivalis* infection. Cytokines were measured using the CBA Inflammatory Kit. The data are presented as means ± standard deviations (SD), and corresponding *p*-values (*⁣*^*∗*^*p* < 0.05; *⁣*^*∗∗∗*^*p* < 0.001) are provided to indicate the statistical significance of the differences observed.

Supporting Information 6 Figure S5: Differentiation and activation of lymphocyte B cell line (A20) in response to stimulation of the lymphocyte. The B cell line was subjected to various stimulations, including medium only, indoxyl sulfate (IS), lipopolysaccharide (LPS), and LPS in combination with IS. The population of Pax5+ cells was selected as the subset representing cells undergoing differentiation and activation. In the LPS + IS group, we observed a reduction in the selected double-positive cell populations (as indicated in the figure) compared to the LPS group. This finding suggests functional impairment of B cells in response to uremic conditions.
