# Supplementary material for: Bidirectional Interaction Between Chronic Kidney Disease and Porphyromonas gingivalis Infection Drives Inflammation and Immune Dysfunction
Source: J Immunol Res. 2025 Apr 17;2025:8355738. doi: 10.1155/jimr/8355738 (PMC12021489; doi:10.1155/jimr/8355738)
Supplement: Supporting Information 1 — Figure S1: CKD did not affect inflammatory response to P. gingivalis in vivo. Strain W83 was injected into the subcutaneous chambers in mice. (A) First, we evaluated the survival and health status of the mice. Morbidity and mortality rates were monitored daily and compared for all the groups after bacterial infection. (B) The levels of live P. gingivalis in chamber fluids did not differ significantly. (C) The body mass was monitored and quantified as % of body weight loss at day 7 postinfection. (D) Quantification of serum cytokines in the control group, CKD group (AAI), P. gingivalis-infected group (P.g.), and CKD-P. gingivalis-infected group (AAI + P.g.). (E) Kidney weight was quantified in all four groups of mice. (F) The treated and control kidneys from all groups were stained for interstitial (F4/80+) and glomerular (Mac2+) macrophages. Quantification was performed in Photoshop as percentage of positively stained high-power field (HPF) or quantified by counting as indicated in the figure. Data are shown as means ± SD. ⁣∗p < 0.05, ⁣∗∗p < 0.01, and ⁣∗∗∗p < 0.001. [file 8355738.f1.pdf]

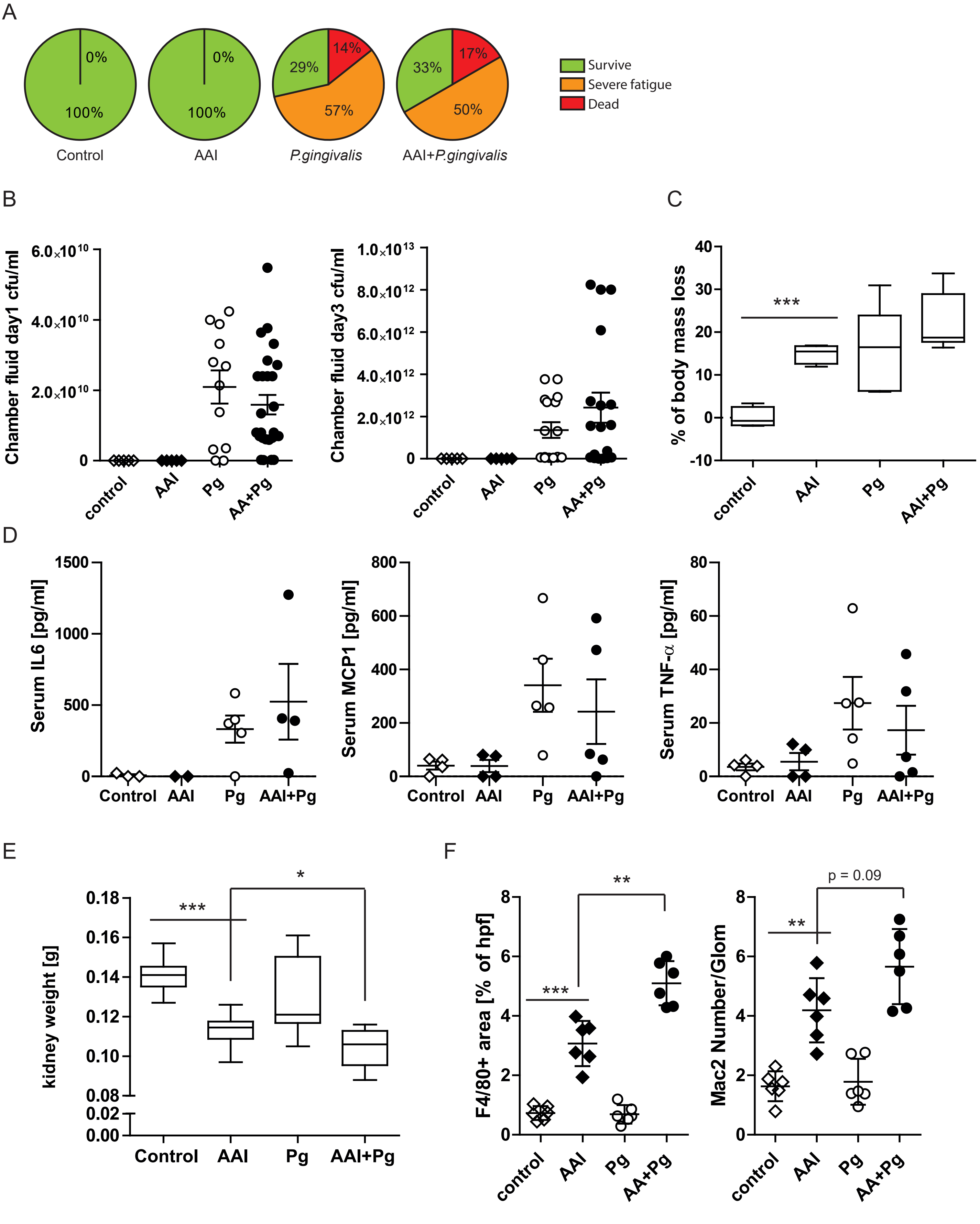

Supplementary Fig. 1 CKD did not affect inflammatory response to *P. gingivalis* in vivo. Strain W83 was injected into subcutaneous chambers in mice. (A) First, we evaluated survival and health status of the mice. Morbidity and mortality rates were monitored daily and compared for all the groups after bacterial infection. (B) Levels of live *P. gingivalis* in chamber fluids did not differ significantly. (C) The body mass was monitored and quantified as % of body weight loss at day 7 post infection. (D) Quantification of serum cytokines in control group, CKD group (AAI), *P. gingivalis*-infected group (P.g.) and CKD-*P.gingivalis* infected group (AAI+P.g.). (E) Kidney weight was quantified in all 4 group of mice. (F) the treated and control kidneys from all groups were stained for interstitial (F4/80+) and glomerular (Mac2+) macrophages. Quantification was performed in Photoshop as percentage of positively stained high-power field (hpf) or quantified by counting as indicated in the figure. Data shown as means ± SD. * $p < 0.05$. ** $p < 0.01$. *** $p < 0.001$.
