# Supplementary material for: Bidirectional Interaction Between Chronic Kidney Disease and Porphyromonas gingivalis Infection Drives Inflammation and Immune Dysfunction
Source: J Immunol Res. 2025 Apr 17;2025:8355738. doi: 10.1155/jimr/8355738 (PMC12021489; doi:10.1155/jimr/8355738)
Supplement: Supporting Information 2 — Figure S2: CKD did not significantly affect response to P. gingivalis in vivo. Strain ATCC 33277 was injected into the subcutaneous chambers in mice. We observed 100% survival and no significant changes in overall health status. (A) We evaluated the body mass for 60 days. (B) The levels of live P. gingivalis in chamber fluids showed prolonged survival of the pathogen within CKD-P. gingivalis-infected group (AAI + P.g.). (C) We did not observe significant changes in MPO activity in chamber fluids between P.g. and AAI + P.g. groups at days 1 and 3. (D) Kidney weights and (E) fibrosis scores showed no differences between CKD group (AAI) and CKD-P. gingivalis-infected group (AAI + P.g.). (F) Kidneys from all groups were stained for T cells (CD3+), interstitial macrophages (F4/80+), proliferation marker (Ki67), and proximal tubuli to illustrate kidney inflammation and regeneration. Quantification was performed in Photoshop as percentage of positively stained high-power field (HPF). Data are shown as means ± SD. ⁣∗p < 0.05 and ⁣∗∗∗p < 0.001. [file 8355738.f2.pdf]

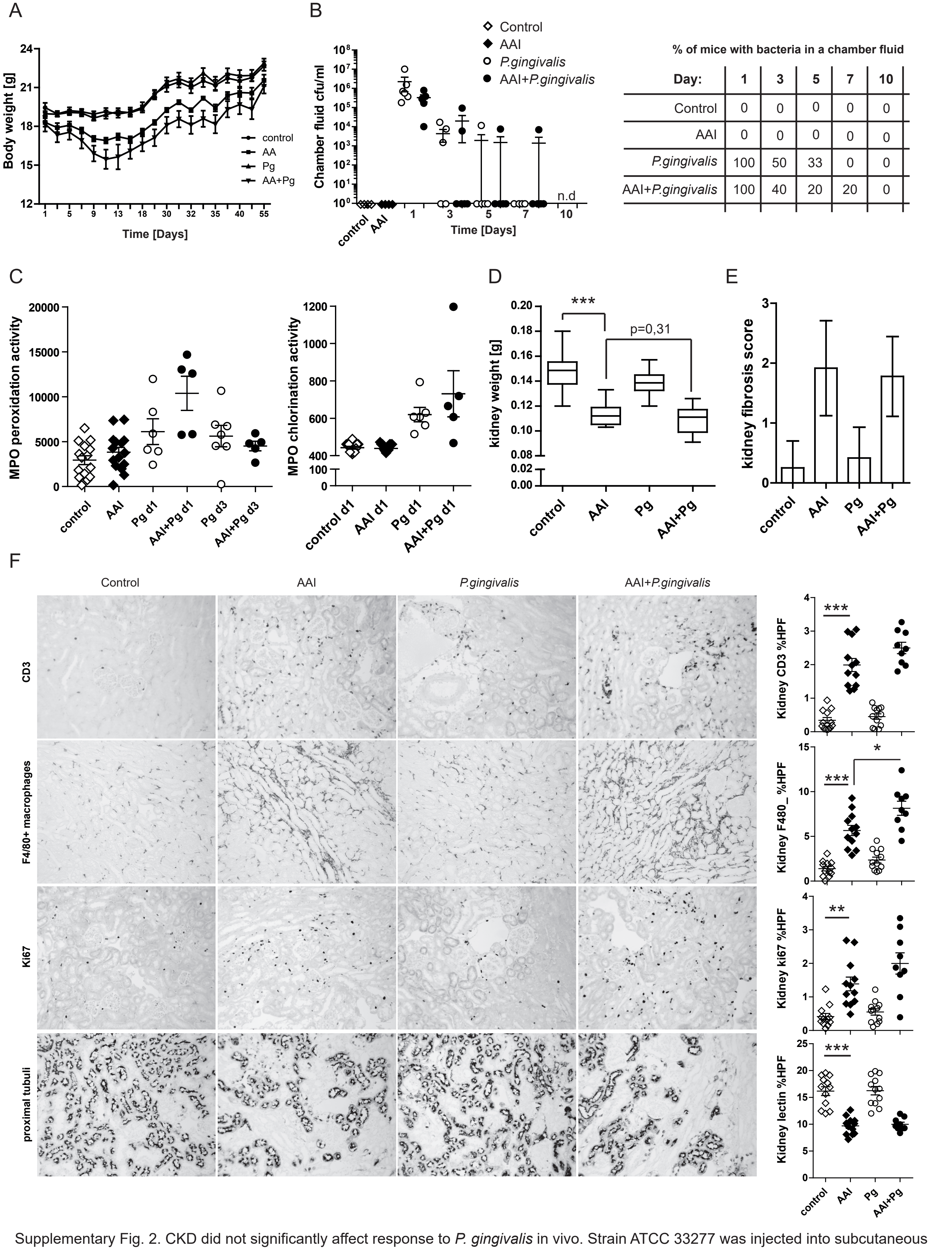

% of mice with bacteria in a chamber fluid

Day:	1	3	5	7	10
Control	0	0	0	0	0
AAI	0	0	0	0	0
P.gingivalis	100	50	33	0	0
AAI+*P.gingivalis*	100	40	20	20	0

Supplementary Fig. 2. CKD did not significantly affect response to *P. gingivalis* in vivo. Strain ATCC 33277 was injected into subcutaneous chambers in mice. We observed 100% survival and no significant changes in overal health status (A) we evaluated body mass for 60 days. (B) Levels of live *P. gingivalis* in chamber fluids showed prolonged survival of the pathogen within CKD-P.gingivalis infected group (AAI+P.g) (C) we did not observe significant changes in MPO activity in chamber fluids between P.g. and AAI+P.g. groups at days (d) 1 and 3. (D) Kidney weights and (E) fibrosis scores showed no differences between CKD group (AAI) and CKD-*P.gingivalis* infected group (AAI+P.g). (F) kidneys from all groups were stained for T-cells (CD3+), interstitial macrophages (F4/80+), proliferation marker (Ki67) and proximal tubuli to illustrate kidney inflammation and regeneration. Quantification was performed in Photoshop as percentage of positively stained high-power field (HPF). Data are shown as means ± SD. * $p < 0.05$. *** $p < 0.001$.
