# Supplementary material for: Bidirectional Interaction Between Chronic Kidney Disease and Porphyromonas gingivalis Infection Drives Inflammation and Immune Dysfunction
Source: J Immunol Res. 2025 Apr 17;2025:8355738. doi: 10.1155/jimr/8355738 (PMC12021489; doi:10.1155/jimr/8355738)
Supplement: Supporting Information 3 — Table S1: Primer list. [file 8355738.f3.pdf]

Table.1. Primer list

Primer name	Accession Nr.	Sequence_Fw	Sequence_Rv
A20	NM_009397	AAGCTCGTGGCTCTGAAAAC	TTCCTCAGGACCAGGTCAGT
Bach2	NM_001109661	GTCGAAAGAGGAAGCTGGACTG	GAGGCAGGAAAAGTTGTCCAGG
Bcl6	NM_009744	AGTTTCTAGGAAAGGCCGGA	ACTAGCGTGCCGGGTAAACT
Bcl10	NM_009740	ACGGAGGAGGATTTGACTGA	TGCACGTAGATGATCAAAATGTC
Ccl2	NM_011333	GCTACAAGAGGATCACCAGCA	GTCTGGACCCATTCCTTCTTG
Ccl5	NM_013653	CCACTTCTTCTCTGGGTTGG	GTGCCCACGTCAAGGAGTAT
Ccl20	NM_016960	GTGGGTTTCACAAGACAGATGGC	CCAGTTCTGCTTTGGATCAGCG
Ccl28	NM_020279	GTTCATGCAGCATCCAGAGAGC	TCTGAGGCTCTCATCCACTGCT
Cd19	NM_009844	GCCACAGCTTTAGATGAAGGCAC	CATCCACCAGTTCTCAACAGCC
Cd86	NM_019388	GATGGACCCCAGATGCACCA	ACGGCAGATATGCAGTCCCA
Cxcl1	NM_008176	ACCCAAACCGAAGTCATAGCC	CTCCGTTACTTGGGGACACC
Cxcl13	NM_018866	CATAGATCGGATTCAAGTTACGCC	GTAACCATTTGGCACGAGGATTC
Cxcr5	NM_007551	ATCGTCCATGCTGTTCACGCCT	CAACCTTGGCAAAGAGGAGTTCC
Foxp3	NM_001199347	TTCATGCATCAGCTCTCCAC	CTGGACACCCATTCCAGACT
Gapdh	NM_001289726	CATGGCCTTCCGTGTTCCTA	CCTGCTTCACCACCTTCTCA
Gata3	NM_001355110	GCCTGCGGACTCTACCATAA	AGGATGTCCCTGCTCTCCTT
Ifng	NM_008337	AGGAACTGGCAAAAGGATGGT	TCATTGAATGCTTGGCGCTG
IL-2	NM_008366	GCGGCATGTTCTGGATTTGACTC	CCACCACAGTTGCTGACTCATC
Il6	NM_031168	TGATGCACTTGCAGAAAACA	ACCAGAGGAAATTTTCAATAGGC
Il10	NM_010548	ACAGCCGGGAAGACAATAACT	CCTGCATTAAGGAGTCGGTTA
IL-12	NM_001159424	TCTTCTCACCGTGCACATCC	TGGCCAAACTGAGGTGGTTT
Il-17	NM_010552	CAGGGAGAGCTTCATCTGTGT	GCTGAGCTTTGAGGGATGAT
Irf4	NM_013674	TGCAAGCTCTTTGACACACA	CAAAGCACAGAGTCACCTGG
Ms4a1	NM_007641	CATTCTGTCGGCGATGCTGATC	TCTCCAGCTGACAGCAGAACCA
Mzb1	NM_027222	AGACCTGCTCTCAGAACTGGCA	CATCACGCTGATTCTTGGCTCTG
Pax5	NM_008782	TGACGCAGGTGTCATCGGTGAG	ATTCGGCACTGGAGACTCCTGA
Prdm1	NM_007548	ACCAAGGAACCTGCTTTTCA	TAGACTTCACCGATGAGGGG
Rorc	NM_001293734	ACAGAGACACCACCGGACAT	GGTGATAACCCCGTAGTGGA
S100a8	NM_013650	CAAGGAAATCACCATGCCCTCTA	ACCATCGCAAGGAACTCCTCGA
Ship	NM_010566	GCTGTTCCGGAATTGTGTTT	GTGAAGAACCTCATGGGGAC
Tank	NM_001164071	GCTTCCAGAATGGGTACGTG	TGGTAGGAATGCCAGCTCTC
T-bet	NM_019507	TCAACCAGCACCAGACAGAG	ATCCTGTAATGGCTTGTGGG
Tlr7	NM_133211	GTGATGCTGTGTGGTTTGTCTGG	CCTTTGTGTGCTCCTGGACCTA
Tnfa	NM_013693	AGGGTCTGGGCCATAGAACT	CCACCACGCTCTTCTGTCTAC
Txlna	NM_001005506	CACCACGTTCAAACAGGAGATGG	CATCTCCAGCAGAGCCTTGTTG
Xbp1	NM_013842	TGGACTCTGACACTGTTGCCTC	TAGACCTCTGGGAGTTCCTCCA
