# Supplementary material for: Bidirectional Interaction Between Chronic Kidney Disease and Porphyromonas gingivalis Infection Drives Inflammation and Immune Dysfunction
Source: J Immunol Res. 2025 Apr 17;2025:8355738. doi: 10.1155/jimr/8355738 (PMC12021489; doi:10.1155/jimr/8355738)
Supplement: Supporting Information 4 — Figure S3: (A) The indican/indoxyl sulfate levels in urine were measured with MAK128 (Sigma) kit at 480 nm. (B) Since indole is a precursor of indoxyl sulfate and is produced as a metabolic intermediate during the breakdown of the amino acid tryptophan by bacteria, we measured the production of indole in P. gingivalis strains used in the experiment; we used uropathogenic E. coli as a positive control. (C) Bacterial colonies grown on agar plates containing Luria–Bertani (LB) and blood agar plate (BAP) medium after inoculating peritoneal fluid. Colonies were counted to assess the microbial load in the peritoneal fluid sample 3 days after induction of kidney injury with AAI. [file 8355738.f4.pdf]

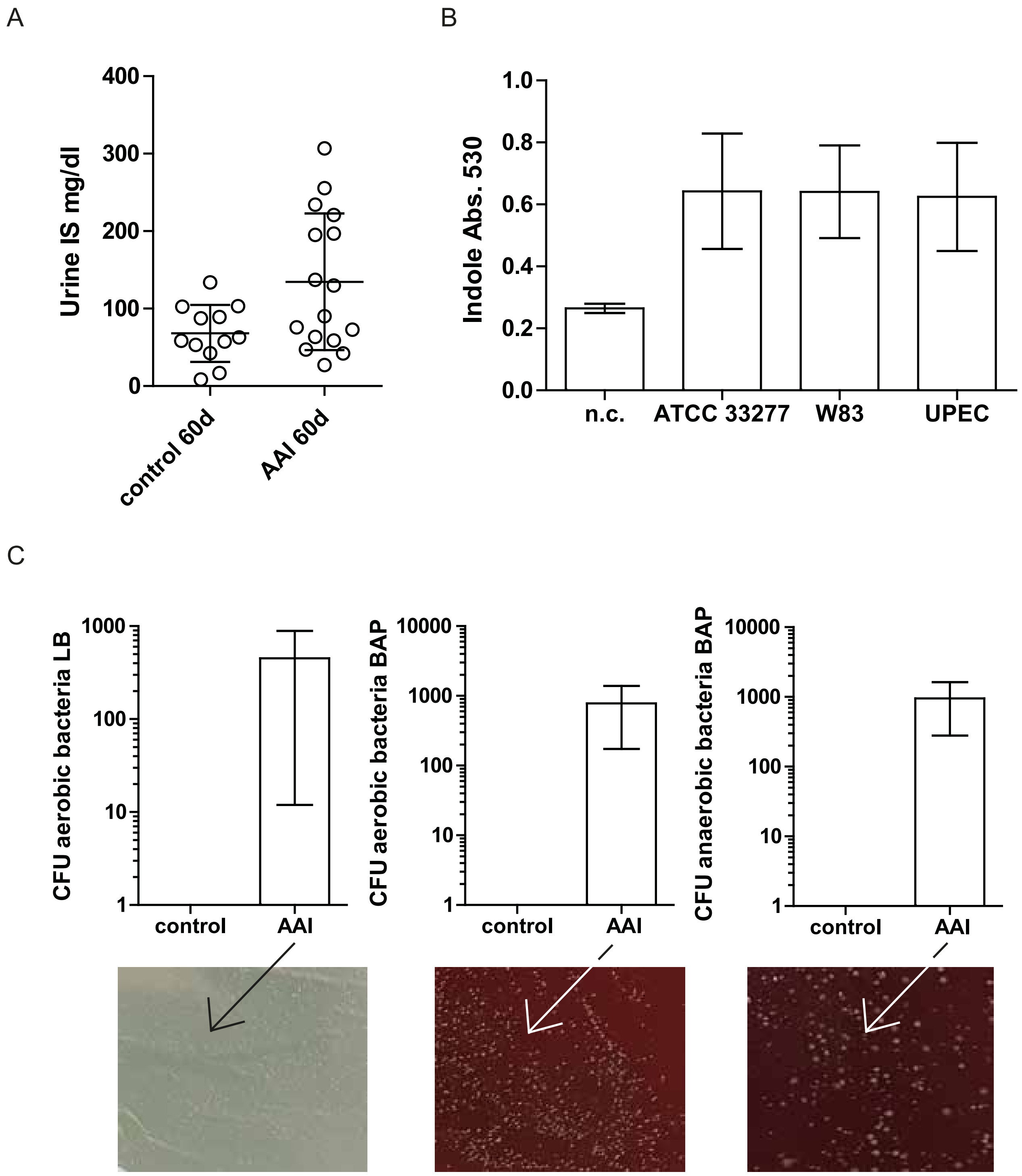

Supplementary Fig. 3. (A) the Indican/Indoxyl Sulfate levels in urine were meassured with MAK128 (Sigma) kit at 480 nm. (B) since indole is a precursor of indoxyl sulfate and is produced as a metabolic intermediate during the breakdown of the amino acid tryptophan by bacteria, we meassured the production of indoole in P. gingivalis strains used in the experiment; we used uropathogenic E. coli as a positive control. (C) Bacterial colonies grown on agar plates containing Luria-Bertani (LB) and Blood Agar Plate (BAP) medium after inoculating peritoneal fluid. Colonies were counted to assess the microbial load in the peritoneal fluid sample 3 days after induction of kidney injury with AAI.
