# Supplementary material for: Bidirectional Interaction Between Chronic Kidney Disease and Porphyromonas gingivalis Infection Drives Inflammation and Immune Dysfunction
Source: J Immunol Res. 2025 Apr 17;2025:8355738. doi: 10.1155/jimr/8355738 (PMC12021489; doi:10.1155/jimr/8355738)
Supplement: Supporting Information 5 — Figure S4: Macrophage cytokine production upon indoxyl sulfate and infection with P. gingivalis. (A) and (B) Bone marrow–derived macrophages (BMDM) were incubated with 60 μg/mL IS for 4 h, infected for 2 h, and washed; cytokine production was measured after 4 and 24 h. We observed a significant increase in the production of cytokines following an infection with P.g. Indoxyl sulfate delays the BMDMs' response to P. gingivalis infection; however, the levels of cytokines in P.g. and P.g. + IS groups at 24 h postinfection are not significantly different. (C) and (D) Human monocyte–derived macrophages (hMDMs) were incubated with 60 μg/mL IS for 4 h, infected for 2 h, and washed; cytokine production was measured after 4 and 24 h. We observed a significant increase in the production of cytokines following an infection with P.g. Indoxyl sulfate does not affect hMDMs' response to P. gingivalis infection. Cytokines were measured using the CBA Inflammatory Kit. The data are presented as means ± standard deviations (SD), and corresponding p-values (⁣∗p < 0.05; ⁣∗∗∗p < 0.001) are provided to indicate the statistical significance of the differences observed. [file 8355738.f5.pdf]

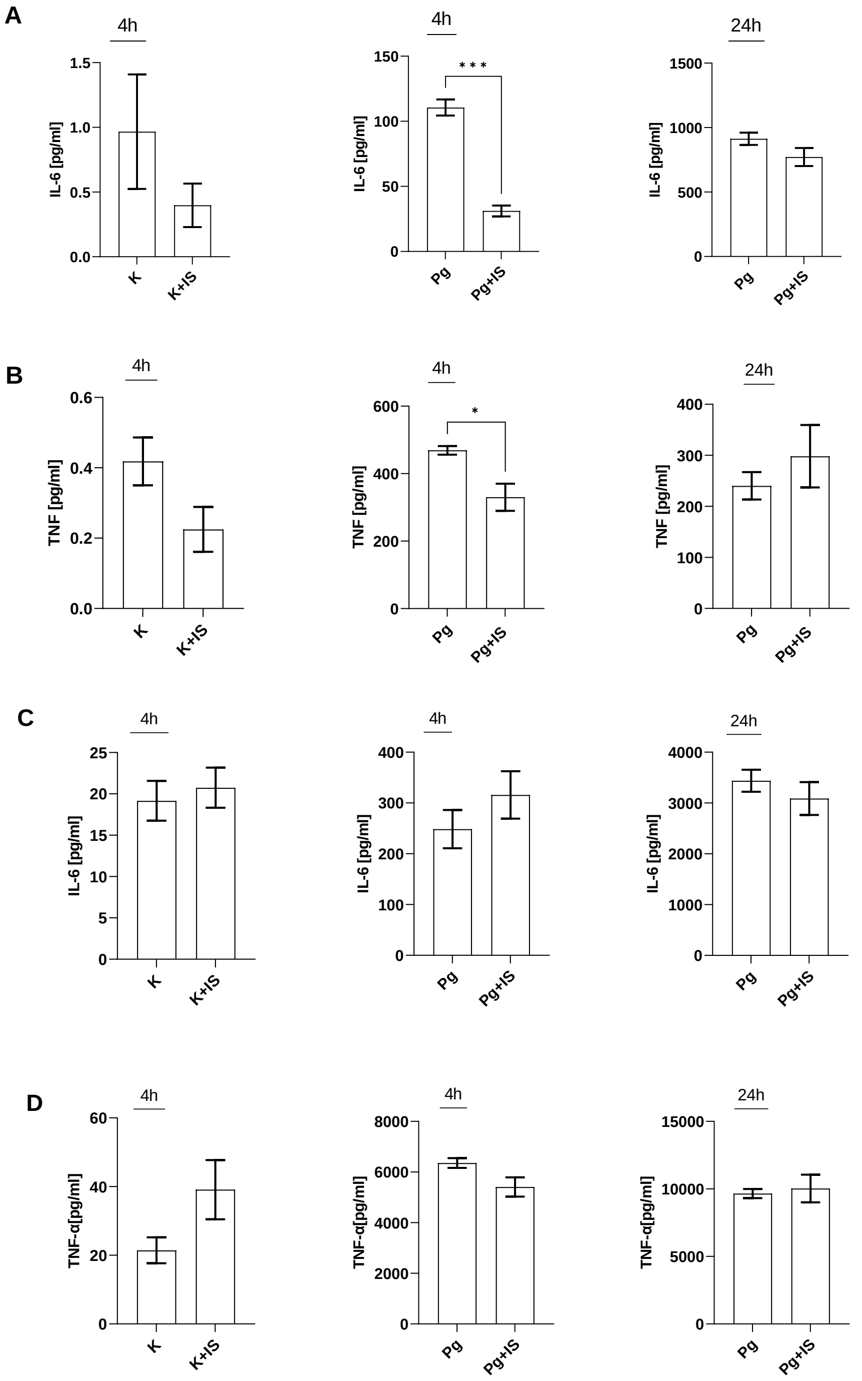

Supplementary Fig. 4 Macrophages´ cytokine production upon indoxyl sulfate and infection with P.gingivalis. (A) and (B): Bone marrow derived macrophages (BMDM) were incubated with 60 µg/ml IS for 4h, infected for 2h, washed, cytokine production was measured after 4h and 24h. We observed a significant increase in the production of cytokines following an infection with P.g. Indoxyl sulfate delay the BMDMs´ response to P. gingivalis infection, however the levels of cytokines in P.g. and P.g.+ IS groups at 24h post infection are not significantly different. (C) and (D) Human derived macrophages (hMDM) were incubated with 60 µg/ml IS for 4h, infected for 2h, washed, cytokine production was measured after 4h and 24h. We observed a significant increase in the production of cytokines following an infection with P.g. Indoxyl sulfate does not affect hMDMs´ response to P. gingivalis infection. Cytokines were measured using CBA Inflammatory Kit. The data is presented as means ± standard deviations (SD), and corresponding p-values (*p < 0.05; ***p < 0.001) are provided to indicate the statistical significance of the differences observed.
