# Supplementary material for: Bidirectional Interaction Between Chronic Kidney Disease and Porphyromonas gingivalis Infection Drives Inflammation and Immune Dysfunction
Source: J Immunol Res. 2025 Apr 17;2025:8355738. doi: 10.1155/jimr/8355738 (PMC12021489; doi:10.1155/jimr/8355738)
Supplement: Supporting Information 6 — Figure S5: Differentiation and activation of lymphocyte B cell line (A20) in response to stimulation of the lymphocyte. The B cell line was subjected to various stimulations, including medium only, indoxyl sulfate (IS), lipopolysaccharide (LPS), and LPS in combination with IS. The population of Pax5+ cells was selected as the subset representing cells undergoing differentiation and activation. In the LPS + IS group, we observed a reduction in the selected double-positive cell populations (as indicated in the figure) compared to the LPS group. This finding suggests functional impairment of B cells in response to uremic conditions. [file 8355738.f6.pdf]

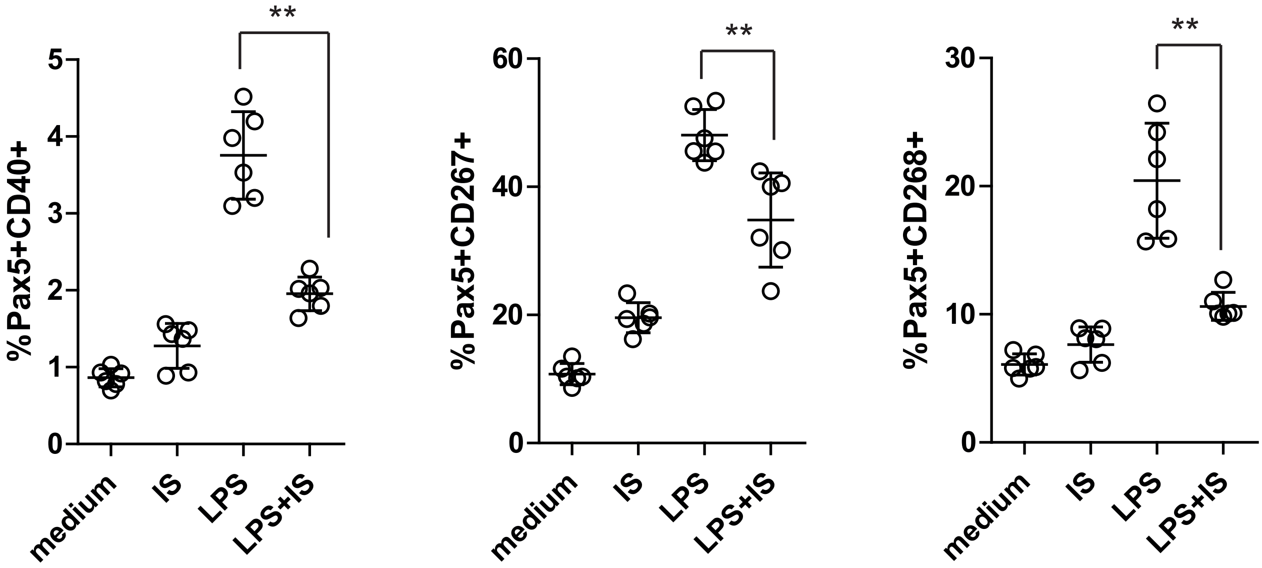

Supplementary Fig. 5. Differentiation and activation of lymphocyte B cell line (A20) in response to stimulation the lymphocyte. B cell line was subjected to various stimulations, including medium only, indoxyl sulfate (IS), lipopolysaccharide (LPS), and LPS in combination with indoxyl sulfate. The population of Pax5+ cells was selected as the subset representing cells undergoing differentiation and activation. In the LPS+IS group, we observed a reduction in the selected double-positive cell populations (as indicated in the figure) compared to the LPS group. This finding suggests functional impairment of B cells in response to uremic conditions.
